# Supplementary material for: Adaptive Changes in the Central Control of Energy Homeostasis Occur in Response to Variations in Energy Status
Source: Int J Mol Sci. 2021 Mar 8;22(5):2728. doi: 10.3390/ijms22052728 (PMC7962960; doi:10.3390/ijms22052728)
Supplement: Supplementary file 1 [file ijms-22-02728-s001.pdf]

METHODS AND MATERIALS

ANIMAL MODELS

All care of animals and procedures were in accordance with the NIH Guide for the Care and Use of Laboratory Animals, and approved by the Western University of Health Sciences' IACUC. Male and female eGFP-POMC mice (18-43g; 55-93days of age) were bred in house or purchased from Jackson Laboratories (Stock #009593; C57BL/6 background). Male and female PACAP-Cre mice (18-43g; 52-144 days of age) were also bred in house or purchased from Jackson Laboratories (Stock #030155; C57BL/6 background) as well. For some experiments, hemizygous eGFP-POMC mice were bred in house with hemizygous PACAP-Cre mice creating a double transgenic eGFP-POMC/ PACAP-Cre mouse model. Pups of 21 days of age were then genotyped using ear snips and standard PCR techniques to detced eGFP and cre amplicons. All animals were kept under a 12:12 hour light/dark cycle (lights on at 06:00-18:00), and were provided food and water ad libitum under constant temperature (25ºC) until they were randomly assigned to one of two dietary conditions (see below).

DIETARY CONDITIONS

eGFP-POMC, PACAP-Cre and double transgenic eGFP-POMC/ PACAP-Cre mice were randomly assigned to either ad libitum or restricted feeding conditions. Those animals under restricted access were given standard rodent chow (Teklad Rodent Diet, Teklad Diets, Madison, WI, USA) from which 18% of the calories were derived from fat, 24% from protein, and 58% from carbohydrates, for six hours (from 8:00-14:00) every day for five days, with an 18-hour fast in between feedings. Water was available ad libitum for these fasted animals.

SURGICAL PROCEDURES

For all experiments, female eGFP-POMC, PACAP-Cre, double transgenic eGFP-POMC/ PACAP-Cre and wildtype (WT) mice were ovariectomized (OVX) while under 2% isoflurane anesthesia five days prior to experimentation. In order to focally inject adeno-associated viral vector (AAV) constructs, male and female PACAP-Cre and double transgenic eGFP-POMC/ PACAP-Cre mice were placed under 2% isoflurane and fixed on a stereotaxic frame (Stoelting, Wood Dale, IL, USA). An incision was made to expose the skull, and a hole was drilled on one or both sides of the mid-sagittal suture so that an injection needle could be slowly lowered into the VMN (coordinates from bregma: AP, -0.6mm; ML, ± 0.3mm; and DV, -5.6mm from dura). Unilateral or bilateral injections of a Cre recombinase-dependent AAV vector containing cation channel rhodopsin-2 (ChR2 {Mattis, 2012 #3152}; AAV1.EF1a.DIO.ChR2 (E123A).YFP.WPRE.jGH; 7.2 x 1012 genomic copies/mL; 300nL total volume; University of Pennsylvania Vector Core; Addgene plasmid #35507) were given over the span of 2 minutes. The injection needle remained in place for 5 minutes before injection and 10 minutes after infusion to allow for diffusion from the tip, and then slowly removed from the brain to reduce inadvertent spread of the virus. Animals were used for experimentation 2-3 weeks after viral injection (1-2 weeks after OVX in females).

The stereotaxic implantation of a guide cannula into the ARC of WT mice was performed similar to that described above. Once anesthetized, an animal was secured on a stereotaxic frame and a midline incision was made through the scalp. A hole was then drilled in the skull, through which a 26-gauge guide cannula (Plastics One, Roanoke, VA, USA) was lowered 1 mm above the ARC (AP, -0.6 mm, ML -0.3 mm, DV -4.9 mm). The guide cannula was fastened in place with C&B Metabond dental cement (Parkell, Edgewood, NY, USA) applied to the surgical field. Finally, a stylet was inserted into the guide cannula to

keep the lumen patent. The animals were allowed to recover for 1 week prior to the start of experimentation. At the time of injection, an injector needle was inserted such that its tip protruded 1 mm below the tip of the guide cannula shaft and into the region of interest.

DRUGS

All drugs were purchased from Tocris Bioscience/ R&D Systems (Minneapolis, MN, USA) unless otherwise stated. For electrophysiological experiments, the Na^+ channel blocker octahydro-12-(hydroxymethyl)-2-imino-5,9:7,10a-dimethano-10aH-[1,3]dioxocino[6,5-d]pyrimidine-4,7,10,11,12-pentol (Tetrodotoxin, TTX) was prepared as a 1mM stock solution in UltraPure H_20, and diluted further with aCSF to the working concentration of 500nM. $PACAP_{1-38}$, the PAC1 receptor agonist, was prepared as a 100μM stock solution in UltraPure H_20, and diluted further with aCSF to the working concentration of 100nM. The PAC1 receptor antagonist, $PACAP_{6-38}$, was prepared as a 200μM stock solution in UltraPure H_2O, and diluted further with aCSF to the working concentration of 200nM. The K^+_{ATP} channel blocker tolbutamide (1-butyl-3-(4-methylphenyl)sulfonylurea) was prepared as a 100mM stock solution in UltraPure H_20, and diluted further with aCSF to the working concentration of 100μM. 2-[[[2-(Tetradecyl)phenyl]amino]carbonyl]benzoic acid (C0X8005), a competitive PTP1B/TCPTP inhibitor, was dissolved in dimethyl sulfoxide (DMSO) to a stock concentration of 20mM, and further diluted with aCSF to a working concentration of 20μM. The AMPK inhibitor 6-[4[2-(1-piperidinyl)ethoxy]phenyl]-3-(4-pyridinyl)-pyrazolo[1,5-a] dorsomorphin dihydrochloride (Compound C) was dissolved in UltraPure H_20 to a stock concentration of 30mM, and further diluted with aCSF to a working concentration of 30μM. The AMPK activator N,N-Dimethylimidodicarbonimidic diamide hydrochloride (metformin), was dissolved in UltraPure H_20 to a stock concentration of 50mM, and further diluted with aCSF to a working concentration of 500μM. The TRPC5 channel blocker, N-(2-Furanylmethyl)-1-(phenylmethyl)-1H-benzimidazol-2-amine (AC1903) was dissolved in UltraPure H_20 to a stock concentration of 30mM, and further diluted with aCSF to a working concentration of 30μM. 1, 3, 5(10)-Estratrien-3, 17β-diol (17β-estradiol; E2; Steraloids, RI, USA) was dissolved in punctilious ethanol to a stock concentration of 1mM, which was further diluted to a working concentration of 100nM.

For all behavioral experiments, $PACAP_{1-38}$ was prepared as a 150μM stock solution by dissolving it in filtered saline, and injected directly into the ARC (30pmol; 0.2 μL). Estradiol benzoate (EB; Steraloids, Newport, RI, USA) was initially prepared as a 1 mg/mL stock solution in punctilious ethanol. A known quantity of this stock solution was added to a volume of sesame oil sufficient to produce a final concentration of 20 μg/kg following evaporation of the ethanol. All aliquots of the stock solutions were stored at either four or -20 °C until needed for experimentation.

HYPOTHALAMIC SLICE PREPARATION

On the day of experimentation, the animal was briefly anesthetized with 32% isoflurane and rapidly decapitated. The brain was removed from the skull and the hypothalamic area was dissected. The hypothalamic block was then mounted on a cutting platform that was secured in a vibratome, filled with ice-cold, oxygenated (95% O2, 5%CO2), sucrose-based cutting solution (NaHCO3 26; dextrose 10, HEPES 10; Sucrose 208; KCl 2; NaH2PO4 1.25; MgSO4 2; CaCl2 1; in mM). Four to five coronal slices (300μm) through the rostrocaudal extent of the ARC were then procured. The slices were transferred to an auxiliary chamber containing oxygenated aCSF at room temperature, and maintained there until the electrophysiological recording.

ELECTROPHYSIOLOGY

Whole-cell patch clamp electrophysiological recordings from ARC neurons using biocytin-filled electrodes were performed in hypothalamic slices prepared from intact male and OVX female eGFP-POMC, PACAP-Cre, and eGFP-POMC/ PACAP-Cre double transgenic mice that were randomly assigned to ad libitum-fed or fasted conditions. During recordings, the slices were maintained in a chamber perfused with warmed (35 °C), oxygenated aCSF in which the CaCl2 concentration raised to 2mM. Artificial CSF and all drugs (diluted with aCSF) were perfused via peristaltic pump at a rate of 1.5 mL/min. Patch electrodes were prepared from borosilicate glass (World Precision Instruments, Sarasota, FL, USA; 1.5 mm OD) pulled on a P-97 Flaming Brown puller (Sutter Instrument Co., Novato, CA, USA), and filled with an internal solution containing the following (in mM): potassium gluconate 128; NaCl 10; MgCl2 1; EGTA 11; HEPES 10; ATP 1; GTP 0.25; 0.5% biocytin; adjusted to a pH of 7.3 with KOH; osmolality: 286-320 mOsm. Electrode resistances varied from 3 to 8 MΩ. Recordings were made on an Olympus BX51 W1 fixed stage microscope outfitted with infrared differential interference contrast (DIC) video imaging. A Multiclamp 700B preamplifier (Molecular Devices) amplified potentials and passed current through the electrode. Membrane currents and voltages underwent analog-digital conversion with a Digidata 1550A interface (Molecular Devices) coupled to pClamp 10.5 software. The access resistance, resting membrane potential (RMP), and input resistance were monitored throughout the course of all recordings. If the access resistance deviated greater than 10% of the original value, the recording was ended. Low-pass filtering of the currents was conducted at a frequency of 2 kHz. The liquid junction potential was calculated to be -10mV, and corrected for during data analysis using pClamp software. All recordings were performed at a holding potential of -60mV.

To determine the postsynaptic effects elicited by bath application of PACAP, we first performed recordings in slices from eGFP-POMC mice. For voltage clamp experiments, baseline ramp I/V relationships (from -110 to -30 mV at a rate of 75 mV per second) were generated in the presence of TTX (500nM). After the baseline ramp I/V, PACAP1-38 (100nM) is added along with TTX, and the membrane current (or voltage) is continuously monitored until a new steady-state value is reached, at which time a second ramp I/V relationship is generated. During the PACAP washout, the membrane current (or potential) is again monitored until it returns to its original baseline level, at which time a final ramp I/V relationship is taken to ensure reversibility of the PACAP-induced effect. For current clamp experiments, the membrane potential and firing rate are monitored from rest in the absence of TTX until new, PACAP-induced steady-state levels are achieved, and then monitored for an additional 10-20 minutes to allow for the return to baseline levels. To determine if these postsynaptic effects are PAC1 receptor- and K^{+}_{ATP} channel-mediated, and negatively modulated by E2, these same recordings were performed in slices pre-treated with 200nM PACAP6-38, 100μM Tolbutamide, 20μM CX08005, 30μM Compound C, 500μM metformin, 30μM AC1903 or 100 nM E_2.

For the optogenetic experiments, recordings were performed in slices from eGFP-POMC/ PACAP-Cre double transgenic and PACAP-Cre mice that were injected with a ChR2-containing viral vector into the VMN 2-3 weeks prior to experimentation. Once PACAP-expressing fibers (visualized with eYFP) impinging on POMC ARC neuron were encountered, functional synaptic connectivity was ascertained by administering high-frequency stimulation (10-ms pulses delivered at 20 Hz for 10 s) from a light-emitting diode (LED) blue light source (470nm) controlled by a variable 2A driver (ThorLabs, Newton, NJ, USA) that directly delivered the light path through the Olympus 40X water-immersion lens. For voltage clamp experiments, baseline ramp I/V relationships were generated in the presence of TTX (500nM). After generating baseline ramp IV's, we would apply the photo-stimulation to generate the slow outward current (in voltage clamp) or hyperpolarization and decrease in firing (in current clamp). Once a new steady-state current (or voltage) was reached, we generated a second ramp I/V, after which the membrane current (or

voltage) was allowed to return to its original baseline level, at which time a final ramp I/V relationship is taken to ensure reversibility of the optogenetic stimulation.

BEHAVIORAL STUDIES

The behavioral studies were conducted using a four-station Comprehensive Lab Animal Monitoring System (CLAMS; Columbus Instruments, Columbus, Ohio, USA) as previously described and validated. Energy intake and energy expenditure such as, O2 consumption and heat production in intact male and OVX female WT mice that were randomly assigned to ad libitum fed or fasting groups. An electronic balance measured the amount of food contained within each food trough and the amount consumed by each animal. Each food trough was equipped with an outer spillage container that effectively mitigated against overestimating the actual amount consumed. At the start of each experiment, animals were allowed to acclimate in their CLAMS chamber over a 3-day period. Each day they were weighed, handled, and returned to their respective chambers. After the 3-day acclimation period, we initiated the 5-day monitoring phase during which the animals were injected each day at 8:00am with either $PACAP_{1-38}$ (30pmol), or its 0.9% saline vehicle (0.2 µL) administered directly into the ARC. OVX female WT mice were injected subcutaneously with either EB (20 µg/kg; s.c.) or its sesame oil vehicle (1 mL/kg; s.c.) every other day over the course of the experiment, starting with acclimation day 2 and through experiment day 5. These fasted mice were placed in the CLAM chambers and monitored during their feeding period from 8:00am to 2:00pm, then weighed and placed back in their home cages with only water accessible. The parameters of energy intake and energy expenditure were continuously written to computer via an A/D converter. Food intake and measures of energy balance were evaluated at 3 and 6 hours after PACAP or vehicle administration.

IMMUNOHISTOCHEMISTRY

Slices from wildtype mice were initially fixed with 4% paraformaldehyde in Sorenson's phosphate buffer (pH 7.4) overnight. They were then immersed for three days in 20% sucrose dissolved in Sorensen's buffer, changed daily, and frozen in tissue-tek embedding medium (miles, inc., elk-hart, in, usa) the next day. We cut coronal sections (20 µm) through the ARC and arc using a cryostat, which we mounted on chilled slides. Next, these sections were washed with 0.1m sodium phosphate buffer (pbs; ph 7.4), and processed overnight with polyclonal antibodies directed against PTP1B (Proteintech, Rosemont, IL, USA; 1:100 dilution) or pAMPK (Invitrogen, Carlsbad, CA, USA; 1:100). This was followed the next day by two 15-minute washes with pbs, and then a two-hour incubation with a biotinylated goat anti-rabbit secondary antibody (Jackson Immunoresearch Laboratories, Inc., West Grove, PA, USA). After another series of three 15-minute washes with Sorensen's buffer, there was a final two-hour overlay with streptavidin-alexa flour (AF)546 (Molecular Probes, Inc., Eugene, OR, USA; 1:600), followed by a final series of three 30-minute washes with Sorensen's and cover slipping the slides. The slides were evaluated using fluorescence immunohistochemistry on a Zeiss axioskop 2 plus microscope (Carl Zeiss, Göttingen, Germany). Number of cells were calculated by counting the number of positively labeled cells in a 400 m x 300 m area in three separate sections per animal.

STATISTICAL ANALYSIS

Comparisons between two groups were made using the Student's t-test. Comparisons made between more than two groups were performed using either the one-way or repeated-measures multifactorial analysis of variance (ANOVA) followed by the Least Significant Difference (LSD) test, or alternatively via the Kruskal-Wallis test followed by median-notched box-and-whisker analysis. Differences were considered statistically significant if the alpha probability was 0.05 or less.
